# Supplementary material for: The genome sequence of Acaena ovalifolia Ruiz & Pav., 1798 (Rosales: Rosaceae)
Source: Wellcome Open Res. 2026 Jun 25;11:386. [Version 1] doi: 10.12688/wellcomeopenres.26810.1 (PMC13555088; doi:10.12688/wellcomeopenres.26810.1)
Supplement: Peer review history

# Reviewer report: Reviewer response for version 1

**DOI:** 10.21956/wellcomeopenres.29521.r160598

**Published:** 2026-08-03

**Authors:** Osamah Alisawi

**Affiliations:** 1 University of Kufa, Kufa, Iraq

This study presents a high-quality chromosome-level dual-haplotype genome assembly of Acaena ovalifolia (Two-spined Acaena), created as part of the Darwin Tree of Life project. The study exceeds standard reference standards and fulfills exceptional technological benchmarks, with an Earth BioGenome Project (EBP) metrics score of 6.C.Q69 for Haplotype 1. The assembly effectively resolves 21 chromosomal pseudomolecules for both haplotypes, providing a large resource of 51,813 protein-coding genes to support evolutionary biology and ecological monitoring. PacBio HiFi long reads (48.10 Gb) with Arima-HiC crosslinking (81.00 Gb) are the gold standard for structural genomic biology. It generates accurate individual reads that can cover complex, recurring genomic landscapes in plants.

  

- The consensus quality (QV) scores are remarkably high (Haplotype 1: 69.0, Haplotype 2: 66.4), representing an extremely low base-calling error rate that far exceeds the standard minimum benchmark of $\geq 40$.
-  Phasing the genome into two distinct parental haplotypes using Hi-C data handles the plant's high heterozygosity (12.23%) elegantly.
- The application of the ASCC decontamination pipeline followed by meticulous manual curation (executing 24 breaks and 53 joins) successfully optimized the final structural metrics, expanding the Scaffold N50 by 0.5%.
- Generating complete mitochondrial (365.03 kb) and plastid (156.05 kb) assemblies alongside the nuclear genome ensures holistic downstream phylogeographic study.
- The BUSCO metrics reveal a highly duplicated single copy ortholog rate of 80.4%. While the paper notes this in passing, this heavily implies an ancestral whole-genome duplication (WGD) or polyploid history within this lineage of Rosaceae. The manuscript leaves this biological phenomenon unexamined.
- **Absence of Biological Interpretation**: As a "Data Note", the manuscript entirely lacks discussion on genome evolution, adaptive features of this invasive neophyte, or comparison to related Rosaceae species.
- **Unexplained Discrepancy in Flow Cytometry**: Flow cytometry calculated the 1C-value genome size to be 0.89 pg (~870 Mb). However, GenomeScope2 k-mer analytics estimated the haploid genome size at 349.95 Mb. Individual haplotypes assembled to ~687 Mb. The text fails to provide a reconciliation or breakdown explaining if this species exhibits atypical polyploidy or massive endopolyploidy to cause these varying estimates.

Despite minor omissions in deeper biological reporting—which falls outside the scope of a standard Data Note—this paper is technically flawless. It provides an indispensable genomic foundation for tracking *Acaena ovalifolia* as it expands its footprint across Britain and Ireland.

Are sufficient details of methods and materials provided to allow replication by others?

Yes

Is the rationale for creating the dataset(s) clearly described?

Yes

Are the datasets clearly presented in a useable and accessible format?

Yes

Are the protocols appropriate and is the work technically sound?

Yes

Reviewer Expertise:

My areas of study include genome assembly, coding and non-coding genomic regions, and endogenous viruses that incorporate in repetitive chromosomal regions.

I confirm that I have read this submission and believe that I have an appropriate level of expertise to confirm that it is of an acceptable scientific standard.

# Reviewer report: Reviewer response for version 1

**DOI:** 10.21956/wellcomeopenres.29521.r161261

**Published:** 2026-07-23

**Authors:** Marco Todesco

**Affiliations:** 1 Michael Smith Laboratories, University of British Columbia, Vancouver, Canada

The article reports the generation of a genome assembly for Two-spined Acaena ( *Acaena ovalifolia*), a South American species that is naturalized in the UK. The genome was assembled by combining long PacBio HiFi reads and Hi-C reads, and annotated with the assistance of RNAseq data.

 The quality of the genome assembly was then assessed using standard metrics (N50s, BUSCO, etc.).

 The resulting diploid genome assembly, which was produced as part of the DToL project, is of high quality. Nonetheless, there is quite a large discrepancy between the GenomeScope estimated genome size (350 Mbp), the flow cytometry-based estimate (870 Mbp), and the final assembly size (687 Mbp). While I am assuming the assembly size is the most reliable of the three, it would be interesting to have a short discussion on why this discrepancy exists (especially given the suspiciously high level of BUSCO duplicates at >80%).

Are sufficient details of methods and materials provided to allow replication by others?

Yes

Is the rationale for creating the dataset(s) clearly described?

Yes

Are the datasets clearly presented in a useable and accessible format?

Yes

Are the protocols appropriate and is the work technically sound?

Yes

Reviewer Expertise:

Plant genomics and genetics, plant adaptation, chromosome structure

I confirm that I have read this submission and believe that I have an appropriate level of expertise to confirm that it is of an acceptable scientific standard.

# Reviewer report: Reviewer response for version 1

**DOI:** 10.21956/wellcomeopenres.29521.r160596

**Published:** 2026-07-21

**Authors:** Rohit Joshi

**Affiliations:** 1 CSIR-Institute of Himalayan Bioresource Technology, Palampur, Himachal Pradesh, India

This data note reports the generation of a high-quality, chromosome-level reference genome for *Acaena ovalifolia* (Two-spined Acaena), a perennial Rosaceae species naturalized in Britain and Ireland. Using PacBio HiFi long-read sequencing, Hi-C scaffolding, and RNA-seq data, the authors assembled two phased haplotypes (~687 Mb each), with over 99% of the primary assembly anchored to 21 chromosomes. The genome annotation identified 51,813 protein-coding genes, and the assembly meets Earth BioGenome Project quality standards.

 1- Very high duplicated BUSCO (80.4%) score , which need to be discussed.

Are sufficient details of methods and materials provided to allow replication by others?

Yes

Is the rationale for creating the dataset(s) clearly described?

Yes

Are the datasets clearly presented in a useable and accessible format?

Yes

Are the protocols appropriate and is the work technically sound?

Yes

Reviewer Expertise:

Functional Genomics

I confirm that I have read this submission and believe that I have an appropriate level of expertise to confirm that it is of an acceptable scientific standard.
